# A Heterogeneous *Codonopsis pilosula* Polysaccharide Preparation Attenuates Doxorubicin-Induced Cardiotoxicity in Mice: Associations with Oxidative Stress, Mitochondrial Function, and Gut Microbiota

**DOI:** 10.3390/ijms27156973

**Published:** 2026-08-03

**Authors:** Mingyang Cui, Yinhua Zhu, Jingyi Qi, Ning Su, Kexin Yuan, Zhixi Wei, Yang Zhang, Yufang Shi, Junzhao Gao, Peng An, Junjie Luo, Yongting Luo

**Affiliations:** Department of Nutrition and Health, China Agricultural University, No. 10 Tianxiu Road, Haidian District, Beijing 100193, China

**Keywords:** *Codonopsis pilosula* polysaccharides, doxorubicin-induced cardiotoxicity, oxidative stress, mitochondrial function, gut microbiota

## Abstract

Doxorubicin-induced cardiotoxicity remains a major limitation of anthracycline chemotherapy and is closely associated with oxidative stress and mitochondrial dysfunction. Here, we investigated whether a commercial *Codonopsis pilosula* polysaccharide preparation (CPP) could attenuate Dox-induced cardiac injury in mice. The preparation was characterized as a glucose-dominant heteropolysaccharide with a broad and heterogeneous molecular-weight distribution dominated by a lower-molecular-weight fraction (89.25% of the integrated chromatographic area; Mw = 1.64 kDa). CPP administration improved ejection fraction and fractional shortening and attenuated myocardial fibrosis and serum markers of cardiac injury. Histological and ultrastructural analyses showed preservation of myocardial architecture and mitochondrial integrity. CPP treatment was accompanied by increased SOD and GSH-PX activities, reduced MDA accumulation, partial recovery of mitochondrial respiratory-chain-related transcripts, and restoration of cardiac ATP content. In addition, 16S rRNA gene sequencing showed that high-dose CPP treatment was associated with partial reversal of Dox-induced gut-microbiota alterations, including increased microbial diversity, reduced *Escherichia-Shigella*, and increased *norank_f__Muribaculaceae* and *Ligilactobacillus*. Collectively, the tested heterogeneous CPP preparation attenuated Dox-induced cardiac injury and was accompanied by improved oxidative-stress indices, preserved mitochondrial structure and function, and changes in gut-microbiota composition.

## 1. Introduction

Doxorubicin (Dox), a member of the anthracycline family, plays a critical role in cancer chemotherapy. Over the past five decades, it has been widely utilized for the treatment of various cancer types [1]. Mechanistically, Dox functions as a DNA topoisomerase II inhibitor. It induces DNA double-strand breaks through direct DNA interaction [2], generation of oxidative stress [3], and reduced ATP production [4], ultimately resulting in tumor cell death. However, long-term or high-dose administration of Dox can lead to the development of cardiotoxicity [5]. Dox-induced cardiotoxicity exhibits a significant dose-dependent characteristic. According to prospective cohort studies and meta-analyses, the incidence of cardiotoxicity increases significantly with rising cumulative doses of Dox. Specifically, the incidence of Dox-related cardiotoxicity reaches 26% (95% CI: 18–34%) at a cumulative dose of 550 mg/m^2^, and the risk further escalates to 48% when the cumulative dose reaches 700 mg/m^2^ [6]. In both in vivo and in vitro experiments, Dox has been shown to induce alterations in mitochondrial biogenesis and energy deficiency in normal cells [6,7,8,9]. In mice with cardiac-specific topoisomerase IIβ (TopIIβ) knockout (KO), DNA damage and cardiotoxicity were prevented—this finding leads to the conclusion that TopIIβ plays a fundamental role in Dox-induced cardiotoxicity [10]. Currently, dexrazoxane is the only drug approved for the treatment of anthracycline-induced cardiotoxicity [11]. Nevertheless, this drug is associated with adverse effects, such as an increased incidence of secondary malignancies and impaired cancer treatment efficacy [12,13]. Therefore, there is an urgent need to identify novel therapeutic agents that can provide long-term cardioprotection against Dox-induced cardiotoxicity without compromising its antitumor efficacy.

Plant chemical and biological studies have demonstrated that carbohydrates are among the primary bioactive components in the roots of *Codonopsis pilosula* and are responsible for its therapeutic effects [14]. *Codonopsis pilosula* polysaccharides (CPP) are major bioactive components extracted from *C. pilosula*. They have been reported to exhibit multiple biological activities, including immunomodulatory effects, antioxidant capacity, gut-microbiota-associated metabolic regulation, and energy metabolism-related benefits [15]. In terms of antioxidant activity, oral administration of CPP to mice (in a D-galactose-induced aging model) increases the activities of superoxide dismutase (SOD) and glutathione peroxidase (GSH-PX) in serum and tissues in a dose-dependent manner while decreasing malondialdehyde (MDA) levels—these effects indicate significant anti-aging activity [16,17]. The gut bacteria can degrade plant polysaccharides to produce bioactive metabolites and transport them to the host system, thereby promoting the human metabolic phenotype, including but not limited to short-chain fatty acids (SCFAs^)^ [18]. Among them, butyrate can directly serve as an energy source for colon cells, while acetate can be used as a substrate for cholesterol and long-chain fatty acid synthesis in the liver [19]. Additionally, 16S rRNA gene sequencing and targeted metabolomics have revealed that gavage administration of CPP to spleen deficiency syndrome mice can improve gut microbiota and optimize energy metabolism-related pathways [20], suggesting that CPP may exert a beneficial effect on mitochondrial function. The monosaccharide composition and glycosidic linkage types of CPP vary across different sources. Their monosaccharide components generally include arabinose, glucose, rhamnose, galactose, mannose, glucuronic acid, and galacturonic acid [15]. Differences in the monosaccharide composition of CPP lead to variations in their stability and bioactivity. Specifically, a higher glucose ratio, relatively smaller particle size, rougher surface morphology, higher molecular weight, and long-chain conformation may promote the formation of a more stable triple-helical structure—a property that enhances their bioactivity [21]. Thus, investigating the physicochemical properties of CPP is a key foundation for elucidating the mechanisms underlying their bioactivity.

However, the effect of CPP on Dox-induced cardiotoxicity and oxidative stress remains unclear. This study aimed to evaluate whether the tested CPP preparation attenuates Dox-induced cardiotoxicity and to characterize the accompanying changes in oxidative-stress indices, mitochondrial function, and gut-microbiota composition.

## 2. Results

### 2.1. Physicochemical and Compositional Characterization of the Codonopsis pilosula Polysaccharide Preparation

**Figure 1:**
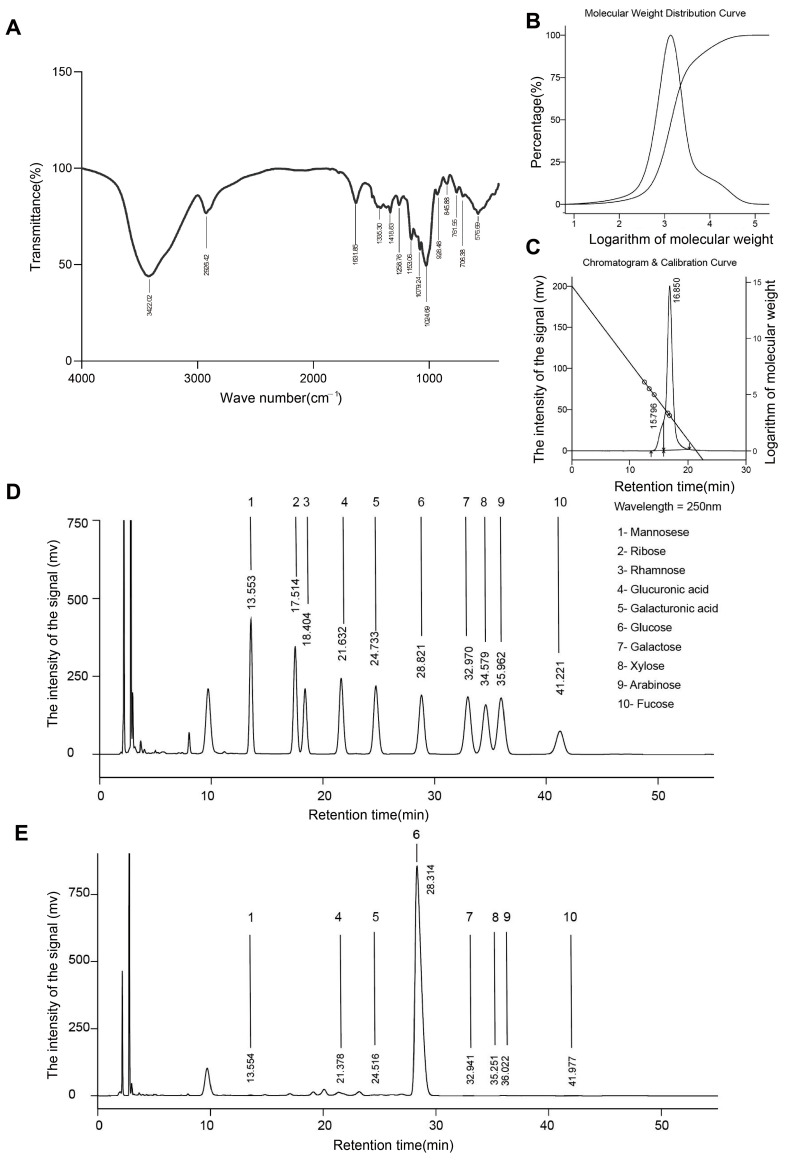
Physicochemical characterization of the *Codonopsis pilosula* polysaccharide preparation (CPP). (**A**) Fourier-transform infrared (FT-IR) spectrum of CPP. (**B**) Molecular-weight distribution curve of CPP analyzed by gel permeation chromatography (GPC). (**C**) Chromatogram and calibration curve of CPP showing the retention times of pullulan standards used for molecular-weight calibration. (**D**) High-performance liquid chromatography (HPLC) chromatogram of standard monosaccharides. (**E**) HPLC chromatogram of CPP hydrolysate.

*Codonopsis pilosula* polysaccharides (CPP; KangRuina Bio Co., Beijing, China, Catalog No. LA2667, stated purity ≥ 98%) were examined for physicochemical characteristics and monosaccharide composition. The FT-IR spectrum showed a broad band at 3422.02 cm^−1^, consistent with O–H stretching, and a band at 2926.42 cm^−1^ attributable to C–H stretching (Figure 1A). Signals at 1418.83 and 1335.30 cm^−1^ were assigned to carboxylate and/or C–H/O–H deformation vibrations, whereas the bands at 1153.06 and 1079.24 cm^−1^ were consistent with C–O–C and C–O vibrations typical of carbohydrate structures. Absorptions at 928.48 and 845.88 cm^−1^ occurred in the anomeric/glycosidic vibration region; however, FT-IR alone does not establish the exact glycosidic-linkage pattern or anomeric configuration [22]. HPLC of the PMP-derivatized hydrolysate detected eight monosaccharides by comparison with authentic standards: mannose, glucuronic acid, galacturonic acid, glucose, galactose, xylose, arabinose, and fucose (Figure 1D,E). Glucose generated the largest chromatographic peak under the analytical conditions.

GPC/SEC analysis showed two integrated molecular-weight fractions (Figure 1B,C). The first, higher-molecular-weight fraction had an apex retention time of 15.796 min and a peak-apex apparent molecular weight of 7.118 kDa. It accounted for 10.75% of the integrated chromatographic area and had Mn = 13.417 kDa, Mw = 18.211 kDa, and Mw/Mn = 1.357. The second, lower-molecular-weight fraction had an apex retention time of 16.850 min and a peak-apex apparent molecular weight of 1.351 kDa. This fraction accounted for 89.25% of the integrated area and had Mn = 0.620 kDa, Mw = 1.639 kDa, and Mw/Mn = 2.645. For the total molecular-weight distribution, Mn, Mw, and Mz were 0.690, 3.420, and 16.790 kDa, respectively, with an overall Mw/Mn of 4.954. Thus, CPP displayed a broad and heterogeneous molecular-weight distribution dominated by the lower-molecular-weight fraction.

### 2.2. CPP Attenuated Doxorubicin-Induced Cardiotoxicity

**Figure 2:**
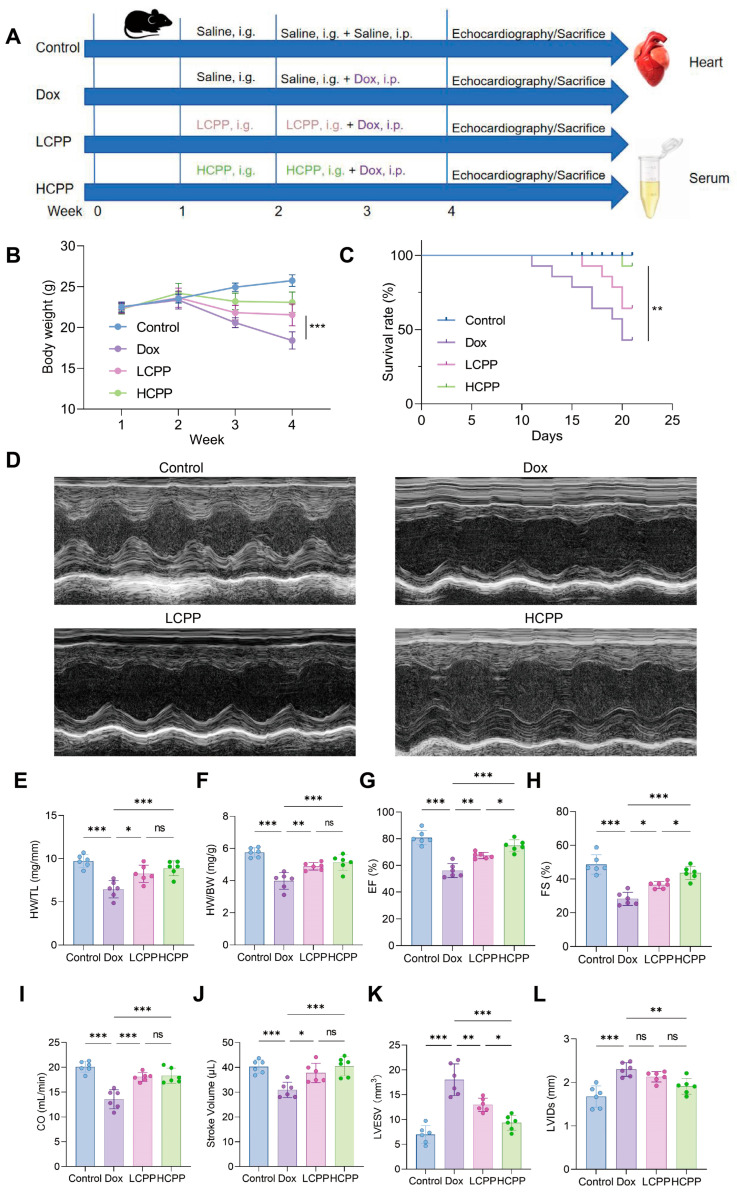
CPP attenuated doxorubicin-induced cardiac dysfunction in mice. (**A**) Experimental timeline and treatment schedule. (**B**) Changes in body weight during the 4-week intervention period. (**C**) Kaplan–Meier survival curves; for each group, *n* = 14. (**D**) Representative echocardiographic M-mode images. (**E**–**L**) Quantitative analysis of cardiac morphometric and functional parameters, including heart weight/tibia length (HW/TL) (**E**), heart weight/body weight (HW/BW) (**F**), ejection fraction (EF) (**G**), fractional shortening (FS) (**H**), cardiac output (CO) (**I**), stroke volume (SV) (**J**), left ventricular end-systolic volume (LVESV) (**K**), and left ventricular internal dimension at end-systole (LVIDs) (**L**); for each group, *n* = 6. For panels (**E**–**L**), group differences were analyzed using one-way analysis of variance (ANOVA), followed by Tukey’s multiple-comparisons test. Survival curves in panel C were analyzed using the Kaplan–Meier method with the log-rank test. Data are presented as mean ± SD. * *p* < 0.05, ** *p* < 0.01, *** *p* < 0.001; ns, not significant.

To evaluate whether CPP could mitigate Dox-induced cardiotoxicity, we conducted animal experiments (Figure 2A). During the intervention, body weight and survival rates of mice were recorded (Figure 2B,C). By week four, Dox-treated mice exhibited marked weight loss, whereas CPP administration mitigated this effect in a dose-dependent manner. Cardiac weight-to-body weight ratio (Figure 2F) and cardiac weight-to-tibia length ratio (Figure 2E) significantly increased following CPP intervention, indicating restoration of relative heart size. Survival analysis further confirmed that CPP enhanced survival rates in Dox-induced mice (Figure 2C).

Echocardiographic assessment at the end of the intervention revealed that CPP treatment significantly alleviated Dox-induced impairments. Specifically, CPP improved ventricular wall motion, partially restoring myocardial systolic and diastolic functions (Figure 2D). Key cardiac-function indices, including ejection fraction (EF) and fractional shortening (FS), showed notable improvement following CPP treatment, with the high-dose group (300 mg/kg/day) outperforming the low-dose group (100 mg/kg/day) (Figure 2G,H). Additionally, CPP significantly reversed the reductions in cardiac output (CO) and stroke volume (SV) induced by Dox (Figure 2I,J). Other parameters, such as left ventricular end-systolic volume (LVESV) and left ventricular internal dimension in systole (LVIDs), were significantly reduced following CPP intervention (Figure 2K,L). These findings indicate that CPP effectively prevents Dox-induced cardiotoxicity.

### 2.3. CPP Alleviated Doxorubicin-Induced Cardiac Injury and Oxidative Stress

**Figure 3:**
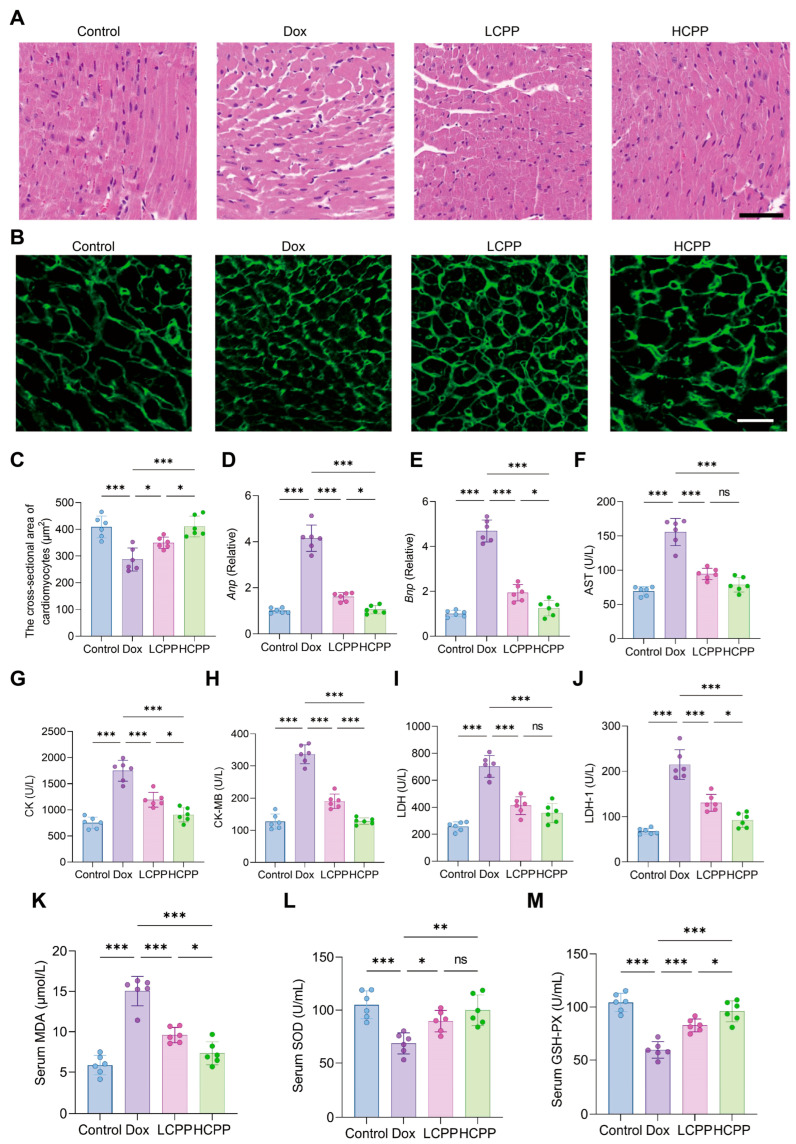
CPP ameliorated doxorubicin-induced cardiac pathological damage and biochemical dysfunction. (**A**) Hematoxylin–eosin (H&E) staining of heart. Scale bar = 20 μm. (**B**) Wheat germ agglutinin (WGA) staining of heart. Scale bar = 20 μm. (**C**) Quantitative analysis of cardiomyocyte cross-sectional area. (**D**,**E**) mRNA expression of cardiac dysfunction markers *ANP* (**D**) and *BNP* (**E**). (**F**–**J**) Serum biochemical indices, including AST (**F**), CK (**G**), CK-MB (**H**), LDH (**I**), and LDH-1 (**J**). (**K**–**M**) Serum levels of oxidative-stress markers, including MDA (**K**), SOD (**L**), and GSH-PX (**M**). For each group, *n* = 6. LCPP, low-dose CPP (100 mg/kg/day); HCPP, high-dose CPP (300 mg/kg/day). One-way analysis of variance (ANOVA) was used to compare the group differences. Significant differences between groups were indicated by *, where * indicates *p* < 0.05; ** indicates *p* < 0.01; and *** indicates *p* < 0.001; ns indicates no significant difference.

To further investigate the protective effects of CPP, cardiac and serum samples were analyzed. Hematoxylin–eosin (HE) staining revealed that Dox induced disorganized myocardial cell arrangement, enlarged intercellular spaces, and structural damage in cardiomyocytes. CPP treatment restored myocardial cell alignment, reduced intercellular spaces, and preserved cellular morphology, approximating the control group (Figure 3A). WGA staining delineated cardiomyocyte borders and showed that Dox reduced cardiomyocyte cross-sectional area. CPP treatment partially restored the cross-sectional area toward control levels, with a more pronounced effect in the high-dose group (Figure 3B,C).

At the molecular level, CPP significantly suppressed mRNA expression of *ANP* and *BNP*, markers of cardiac dysfunction, compared to the Dox group (Figure 3D,E). Serum analysis revealed that Dox significantly elevated levels of LDH, LDH-1, AST, CK, and CK-MB. Low-dose CPP partially reduced these markers, while high-dose CPP exhibited more pronounced effects (Figure 3F,J). These results indicate that CPP mitigates Dox-induced myocardial damage and prevents pathological progression.

Dox-induced cardiotoxicity is closely associated with oxidative stress. MDA, a marker of lipid peroxidation [23], reflects oxidative-stress levels, while SOD and GSH-PX are key antioxidant enzymes [24]. Serum analysis showed that Dox elevated MDA levels and reduced SOD and GSH-PX activity (Figure 3K–M). Both low- and high-dose CPP treatments mitigated oxidative stress, lowering MDA levels and increasing SOD and GSH-PX activity, with high-dose CPP demonstrating superior effects (Figure 3K–M).

### 2.4. CPP Attenuated Doxorubicin-Induced Myocardial Fibrosis

**Figure 4:**
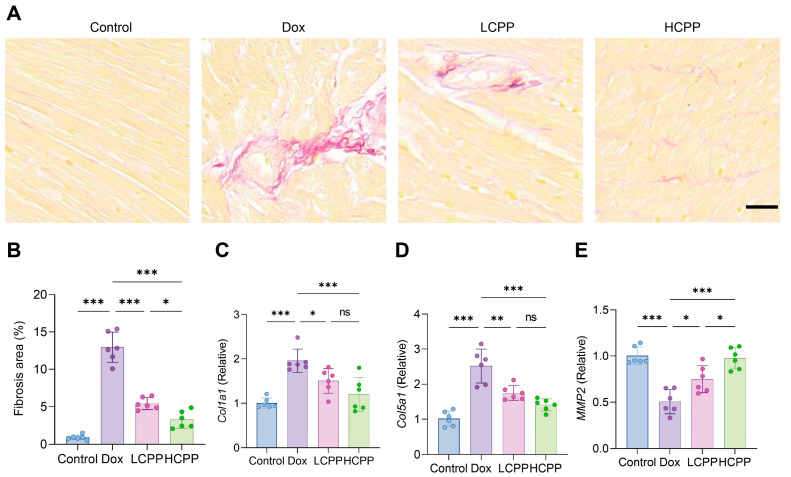
CPP reduced doxorubicin-induced myocardial fibrosis. (**A**) Sirius Red staining of cardiac tissue. Scale bar = 50 μm. (**B**) Quantitative analysis of fibrotic area (%). (**C**–**E**) Relative mRNA expression levels of collagen-related genes *Col1a1* (**C**), *Col5a1* (**D**), and *MMP-2* (**E**). For each group, *n* = 6. One-way analysis of variance (ANOVA) was used to compare the group differences. Significant differences between groups were indicated by *, where * indicates *p* < 0.05; ** indicates *p* < 0.01; and *** indicates *p* < 0.001; ns indicates no significant difference.

The anti-fibrotic effects of CPP were evaluated using Sirius Red staining of cardiac sections [25]. Dox treatment resulted in substantial collagen fiber deposition, characterized by irregular and disorganized red-stained fibers, indicating significant myocardial fibrosis. Low-dose CPP treatment reduced collagen deposition, partially alleviating fibrosis, whereas high-dose CPP further minimized collagen accumulation, closely resembling the control group (Figure 4A,B).

Gene expression analysis revealed that Dox significantly upregulated *Col5a1* and *Col1a1* (encoding type I and type V collagen, respectively) and inhibited *MMP-2* (a collagen-degrading enzyme). CPP intervention reversed these effects, reducing collagen gene expression and restoring *MMP-2* levels, with high-dose CPP demonstrating superior efficacy (Figure 4C–E). These findings highlight CPP’s ability to prevent Dox-induced myocardial fibrosis.

### 2.5. CPP Protected Against Mitochondrial Damage Induced by Doxorubicin

**Figure 5:**
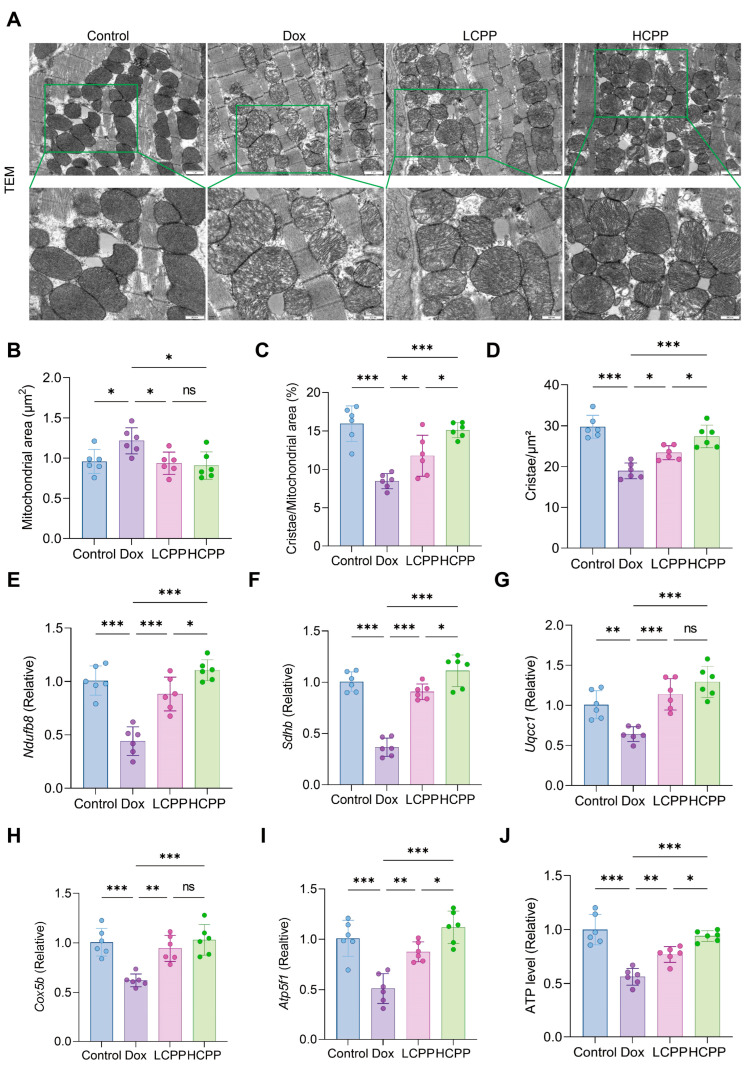
CPP preserved mitochondrial structure and function in doxorubicin-treated mice. (**A**) Representative transmission electron microscopy (TEM) images. Scale bar = 1 μm (upper), 500 nm (lower). (**B**–**D**) Quantitative analysis of mitochondrial area, cristae/mitochondrial area ratio, and cristae density. (**E**–**I**) Relative mRNA expression levels of mitochondrial respiratory-chain complex genes *Ndufb8* (**E**), *Sdhb* (**F**), *Uqcrc1* (**G**), *Cox5b* (**H**), and *Atp5f1* (**I**). (**J**) Determination of the relative content of ATP in cardiac tissue. For each group, n = 6. One-way analysis of variance (ANOVA) was used to compare the group differences. Significant differences between groups were indicated by *, where * indicates *p* < 0.05; ** indicates *p* < 0.01; and *** indicates *p* < 0.001; ns indicates no significant difference.

Excessive Dox exposure has been reported to disrupt mitochondrial biogenesis and energy production, contributing to cardiovascular damage [6], and mitochondria can serve as targets for treating cardiovascular diseases with natural products [23,26]. Here, we assessed mitochondrial ultrastructure using transmission electron microscopy (TEM, Hitachi, Tokyo, Japan) at 5000× and 10,000× magnifications. Dox caused mitochondrial swelling, deformation, and irregular cristae arrangement, with instances of cristae rupture and dissolution, indicating severe mitochondrial damage (Figure 5A). Both low- and high-dose CPP treatments preserved mitochondrial ultrastructure, restoring cristae integrity and approximating the control group (Figure 5A).

Quantitative analysis showed that Dox significantly increased mitochondrial area, reflecting swelling, while CPP treatment reduced mitochondrial area, ameliorating swelling (Figure 5B). The cristae-to-mitochondrial area ratio and cristae density, which reflect cristae organization, were reduced in the Dox group. CPP treatment restored these parameters, with high-dose CPP providing superior protection compared to low-dose CPP (Figure 5C,D). Additionally, CPP partially restored the mRNA levels of genes related to mitochondrial respiratory-chain complexes and ATP synthase (Figure 5E–I). Direct ATP content measurements in fresh cardiac tissue revealed that CPP significantly restored ATP levels, with high-dose CPP showing greater efficacy than low-dose CPP (Figure 5J). These findings indicate that CPP treatment was accompanied by preservation of mitochondrial ultrastructure, partial recovery of respiratory-chain-related transcripts, and restoration of cardiac ATP content.

### 2.6. Impact of Polysaccharide Intervention on Gut-Microbiota Diversity

**Figure 6:**
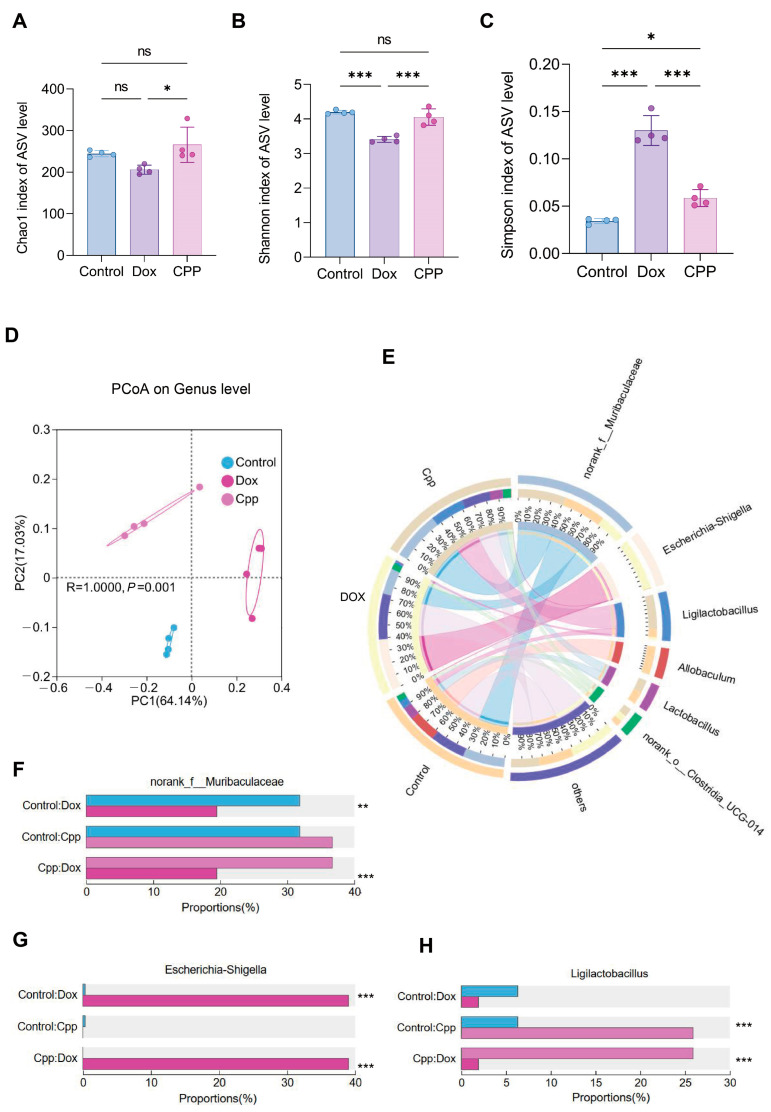
CPP restored gut-microbiota diversity and altered gut-microbial composition in doxorubicin-treated mice. (**A**–**C**) Alpha diversity of the gut microbiota, including the Chao1 index (**A**), Shannon index (**B**), and Simpson index (**C**). Group differences were analyzed using the Kruskal–Wallis test, followed by Dunn’s multiple-comparisons test. (**D**) Principal coordinates analysis (PCoA) based on Bray–Curtis distances at the genus level; differences in community structure among groups were assessed by PERMANOVA. (**E**) Relative abundance of the top genera in each group. (**F**–**H**) Relative abundances of *norank_f__Muribaculaceae* (**F**), *Escherichia-Shigella* (**G**), and *Ligilactobacillus* (**H**). For each group, n = 4. Group differences were analyzed using the Kruskal–Wallis test, followed by Dunn’s multiple-comparisons test. Data are presented as median with interquartile range. * *p* < 0.05, ** *p* < 0.01, *** *p* < 0.001; ns, not significant.

Because the high-dose CPP group showed the most pronounced overall improvements in selected cardiac, oxidative-stress, and mitochondrial outcomes, the exploratory 16S rRNA gene-sequencing analysis focused on the Control, Dox, and HCPP groups. It was reported that polysaccharides also have the function of regulating the gut microbiota, and the gut microbiota can also affect heart health [27]. We used the 16S rRNA gene-sequencing technology to assess the impact of CPP intervention on the diversity of the gut microbiota. The alpha diversity demonstrated that Dox significantly reduced Shannon diversity (Figure 6B) and increased the Simpson dominance index (Figure 6C), whereas CPP shifted both indices toward control levels. Chao1 richness did not differ significantly between the control and Dox groups but was higher in the CPP group than in the Dox group (Figure 6A). Principal coordinates analysis (PCoA) at the genus level (Figure 6D) further confirmed these results, showing clear separation between the Dox and CPP groups. The three groups (Control, Dox, and CPP) were distinctly clustered, with the Dox group being notably separated from the Control and CPP groups, indicating significant alterations in the gut-microbiota structure caused by Dox treatment.

Among the dominant genera, *norank_f__Muribaculaceae*, *Escherichia-Shigella*, and *Ligilactobacillus* showed marked changes across groups (Figure 6E). Dox treatment led to changes in the gut-microbiota composition of mice, with a significant increase in the abundance of the opportunistic pathogen Escherichia-Shigella, while the abundance of beneficial bacteria *norank_f__Muribaculaceae* and *Ligilactobacillus* decreased. After CPP intervention, the abundance of *Escherichia-Shigella* decreased to a level comparable to the control group. Notably, the two most abundant beneficial bacteria, *norank_f__Muribaculaceae* and *Ligilactobacillus*, were well restored, especially *Ligilactobacillus*, which showed significantly higher abundance after polysaccharide intervention compared to the control group (Figure 6F–H).

### 2.7. Correlation Analysis Between Gut Microbiota and Cardiotoxicity-Related Indicators

**Figure 7:**
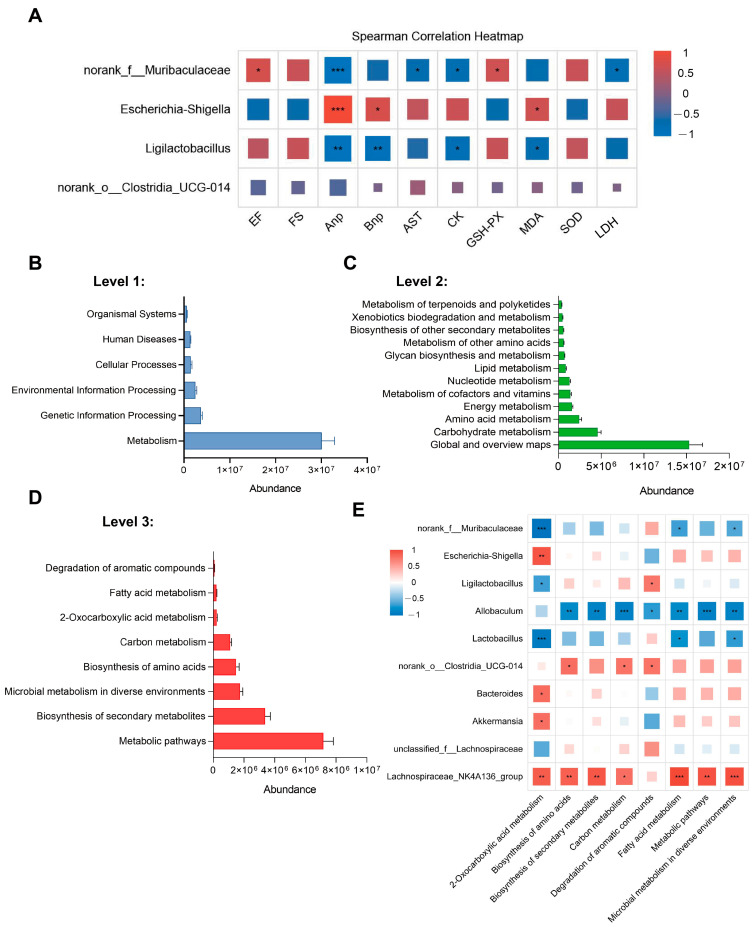
Correlation analysis of gut microbiota with cardiac indicators and predicted microbial functions. (**A**) Heatmap showing correlations between differential gut genera and cardiac/oxidative-stress indices. Correlations were evaluated using Spearman’s rank correlation analysis. (**B**–**D**) Predicted microbial functional pathways based on PICRUSt analysis at KEGG level 1 (**B**), level 2 (**C**), and level 3 (**D**). (**E**) Heatmap showing correlations between differential bacterial genera and KEGG level 3 pathways, analyzed using Spearman’s rank correlation analysis. For each group, *n* = 4. Red indicates positive correlation and blue indicates negative correlation. * *p* < 0.05, ** *p* < 0.01, *** *p* < 0.001.

To explore the relationship between gut-microbiota changes and cardiac function, a Spearman correlation analysis was conducted between the relative abundance of specific gut bacteria and key cardiac-function indicators, including ejection fraction (EF); fractional shortening (FS); cardiotoxicity-related indicators, including *ANP* and *BNP;* and oxidative-stress markers, such as MDA, SOD, and GSH-PX. Specifically, the abundance of *Escherichia-Shigella* was strongly positively correlated with cardiotoxicity-related indicators *ANP* and *BNP* and the oxidative-stress marker MDA, suggesting that higher levels of this genus are associated with poorer cardiac function. Conversely, *norank_f__Muribaculaceae* showed positive correlations with EF and GSH-PX levels, whereas it was negatively correlated with the cardiotoxicity-related indicators *ANP*, AST, and CK. Similarly, the abundance of *Ligilactobacillus* was significantly associated with the levels of the cardiotoxicity markers *ANP*, *BNP*, and CK, as well as with the oxidative-stress marker MDA. In contrast, changes in the abundance of *norank_o_Clostridia_UCG-014* before and after treatment appeared to show no significant correlation with any of the measured indicators (Figure 7A).

Subsequently, KEGG pathway prediction and correlation analyses were performed based on the 16S rRNA sequencing data. At KEGG level 1, the predicted functions were mainly assigned to metabolism, genetic information processing, environmental information processing, and cellular processes (Figure 7B). At KEGG level 2, global and overview maps represented the most abundant functional category, followed by carbohydrate metabolism, amino acid metabolism, and energy metabolism (Figure 7C). At KEGG level 3, metabolic pathways exhibited the highest relative abundance, followed by biosynthesis of secondary metabolites, microbial metabolism in diverse environments, biosynthesis of amino acids, and carbon metabolism (Figure 7D). Furthermore, correlation analysis showed that differential bacterial genera were significantly associated with several KEGG level 3 pathways, suggesting a close relationship between microbial compositional changes and functional alterations after CPP treatment (Figure 7E).

These findings indicate that changes in gut-microbiota composition were associated with cardiac and oxidative-stress indices after CPP treatment.

## 3. Discussion

### 3.1. Cardioprotective Effects and Dose-Related Responses of the CPP Preparation

Here, we characterized the physicochemical features and biological effects of CPP in a mouse model of Dox-induced cardiotoxicity. FT-IR and monosaccharide profiling supported classification of the commercial material as a glucose-dominant heteropolysaccharide preparation, whereas GPC/SEC revealed a broad, heterogeneous molecular-weight distribution dominated by a lower-molecular-weight fraction. In vivo, CPP improved cardiac-functional indices, including ejection fraction, fractional shortening, and cardiac output, and attenuated pathological injury, fibrosis-related changes, and mitochondrial abnormalities. CPP treatment was also accompanied by changes in gut-microbiota diversity and composition.

Although both low and high doses of CPP significantly alleviated Dox-induced cardiac injury, not all parameters differed between the two doses. Indicators such as stroke volume, LVIDs, serum AST and LDH, and SOD activity showed no significant difference between groups (*p* ≥ 0.05). However, in key functional and biochemical markers—including EF, FS, serum CK, mitochondrial cristae density, ATP content, MDA, and GSH-PX—the high-dose group exhibited significantly greater improvement than the low-dose group (*p* < 0.05), indicating a clear dose-dependent enhancement of cardioprotection.

### 3.2. Physicochemical Heterogeneity of the CPP Preparation and Implications for Bioactivity

CPP did not behave as a single, molecularly uniform polysaccharide species. The major lower-molecular-weight GPC/SEC fraction represented 89.25% of the integrated chromatographic area and had Mw = 1.639 kDa, whereas the minor higher-molecular-weight fraction represented 10.75% and had Mw = 18.211 kDa. The broad overall dispersity (Mw/Mn = 4.954) further indicates substantial molecular-weight heterogeneity. These values are apparent molecular weights derived from pullulan calibration and should not be interpreted as absolute masses or as evidence for a defined molecular structure. Moreover, the present unfractionated animal experiment cannot determine which molecular-weight fraction accounts for the observed biological effects. Accordingly, enhanced bioavailability, tissue penetration, or cardioprotective activity should not be attributed to the lower-molecular-weight fraction without fractionation and direct comparative testing.

### 3.3. Attenuation of Oxidative Stress and Preservation of Mitochondrial Function

The cardioprotective effects observed in this study align with previous reports on the therapeutic potential of natural polysaccharides in cardiovascular diseases. Astragalus polysaccharides (APS) have been reported to inhibit mitochondrial apoptosis pathways by suppressing cytochrome c release and regulating the Bcl-2/Bax ratio, thereby ameliorating diabetic cardiomyopathy. Moreover, APS-based nanoparticles exert protective effects against sepsis-induced cardiac dysfunction by suppressing the TLR4/NF-κB signaling pathway [28,29]. In addition, ginsenosides have been shown to alleviate myocardial infarction by activating the Nrf2/HO-1 and PI3K/Akt signaling pathways, thereby inhibiting cardiomyocyte apoptosis, oxidative stress, and mitochondrial dysfunction [30,31]. Similarly, CPP’s antioxidative effects, evidenced by increased SOD and GSH-PX activity and reduced MDA levels, are consistent with prior findings suggesting that polysaccharides from Codonopsis species modulate redox homeostasis and energy metabolism [32]. Moreover, the restoration of mitochondrial integrity observed under CPP treatment parallels the effects of Lycium barbarum polysaccharides, which preserve mitochondrial morphology and enhance ATP synthesis in hepatocytes [33].

The results can be interpreted within the framework of the oxidative stress–mitochondrial dysfunction theory of Dox-induced cardiotoxicity. According to this model, excessive ROS production damages mitochondrial DNA and respiratory-chain complexes, leading to ATP depletion and cardiac dysfunction [34,35,36]. The ability of CPP to partially restore the transcript levels of genes related to mitochondrial respiratory-chain complexes, together with the recovery of cardiac ATP content, is consistent with improved mitochondrial energy homeostasis. In this context, the Nrf2/HO-1 and AMPK/PGC-1α signaling pathways represent two plausible molecular pathways that warrant further investigation. Nrf2/HO-1 signaling plays an important role in antioxidant defense, and activation of Nrf2 has been shown to attenuate oxidative injury and cardiac dysfunction in experimental models of Dox-induced cardiotoxicity [37]. Meanwhile, AMPK/PGC-1α signaling is closely associated with mitochondrial energy metabolism and biogenesis, and its activation has been reported to alleviate Dox-induced mitochondrial oxidative damage and cardiac injury [38,39]. In the present study, the increased SOD and GSH-PX activities and reduced MDA levels are compatible with enhanced antioxidant defense, whereas the partial recovery of respiratory-chain-related transcripts and ATP content is compatible with improved mitochondrial homeostasis. However, because Nrf2 nuclear translocation, HO-1 expression, AMPK phosphorylation, and PGC-1α-related signaling were not directly assessed, the involvement of these pathways cannot be confirmed and should be regarded as a hypothesis for future investigation. These findings may also be considered in relation to the hormesis theory, which proposes that moderate stressors, such as mild oxidative challenges, can trigger adaptive antioxidant responses [40]. CPP may therefore influence redox homeostasis by enhancing endogenous defense mechanisms, although this interpretation requires direct validation. In addition, from a systems-pharmacology perspective, polysaccharides such as CPP may influence multiple signaling pathways simultaneously, including PI3K/Akt, AMPK, and Nrf2, which could collectively contribute to cardioprotective outcomes. This potential multi-target action is consistent with the interaction model frequently proposed for traditional herbal medicines and natural bioactive products [41,42].

### 3.4. Gut-Microbiota Alterations Associated with CPP Treatment

Notably, the gut-microbiota data add an additional associative dimension to our findings. In the present study, CPP treatment was associated with partial recovery of overall microbial diversity in mice with Dox-induced cardiotoxicity and with changes in microbial community composition, characterized by increased abundances of *norank_f__Muribaculaceae* and *Ligilactobacillus* and a decreased abundance of *Escherichia-Shigella*. Moreover, these compositional changes were correlated with cardiac-function and oxidative-stress indices, supporting an association between gut-microbial composition and host cardiac and redox status. This pattern is broadly consistent with previous evidence showing that doxorubicin induces gut dysbiosis and suggesting that microbiota disturbances may be involved in cardiotoxic progression through inflammatory, metabolic, and redox-related pathways. In addition, fecal microbiota transplantation has been reported to modify the severity of DOX-induced myocardial injury [43,44].

Our results also agree with earlier work on *Codonopsis pilosula* polysaccharides in non-cardiac disease models, in which CPP improved gut-microbial homeostasis and promoted microbiota-dependent metabolic benefits, including SCFA-related effects and optimization of energy metabolism [45]. However, an interesting difference was observed between our study and previous reports: following CPP intervention, several bacterial taxa showed marked changes, particularly the concurrent reduction in *Escherichia-Shigella* and the restoration of *norank_f__Muribaculaceae* and *Ligilactobacillus*. The expansion of *Escherichia-Shigella* is commonly associated with intestinal barrier dysfunction, increased endotoxin exposure, and enhanced inflammatory responses [46]. In contrast, *Muribaculaceae* is widely recognized in mouse studies as a key polysaccharide-utilizing taxon with potential SCFA-related metabolic benefits [47], whereas *Ligilactobacillus*, as a lactic acid-producing lineage, is often considered to exert antioxidant and mucosal protective effects [48]. These compositional shifts are consistent with the previously reported metabolic characteristics of these taxa, although their functional consequences were not directly measured in the present study. Nevertheless, the present 16S rRNA gene-sequencing and correlation analyses cannot determine whether the microbial changes contributed to cardiac improvement or occurred secondary to improvements in cardiac or systemic status. Fecal microbiota transplantation or microbiota-depletion studies will be required to establish causality.

### 3.5. Limitations

Several considerations should be noted when interpreting the present findings. First, only male mice were included, and no pharmacological-positive-control group was used; therefore, sex-dependent responses and the relative efficacy of the CPP preparation compared with an established cardioprotective agent were not evaluated. Second, the gut-microbiota analysis was restricted to the high-dose group and was based on 16S rRNA gene-sequencing and correlation analyses. Thus, neither a dose–response relationship nor a causal role of the microbiota can be established. Third, the tested commercial CPP preparation was molecularly heterogeneous and was not fractionated, preventing identification of the active molecular-weight fraction or structural component. Finally, the intracellular signaling pathways underlying the observed antioxidant and mitochondrial effects were not directly examined.

### 3.6. Conclusions

In this mouse model, the tested commercial, molecularly heterogeneous *Codonopsis pilosula* polysaccharide preparation attenuated Dox-induced cardiac dysfunction, myocardial injury, and fibrosis. These effects were accompanied by improved antioxidant-related indices, preservation of mitochondrial ultrastructure, partial recovery of mitochondrial respiratory-chain-related gene expression, and restoration of cardiac ATP content. High-dose CPP treatment was also associated with partial normalization of Dox-induced gut-microbiota alterations. Collectively, these findings support further investigation of the CPP preparation as a potential cardioprotective intervention.

## 4. Methods and Materials

### 4.1. Animals and Treatments

All animal experimental operations received approval and were conducted according to the guidelines of the Animal Welfare and Ethics Review Committee of China Agricultural University (No. AW82805202-5-01). Male C57BL/6J mice were housed in a specific pathogen-free animal facility of China Agricultural University. The temperature in the animal facility was maintained at 24 ± 2 °C, with a humidity of 55 ± 5%. Mice were placed in individually ventilated, pathogen-free cages. A total of 56 mice were used, with 14 mice in each group. Mice were kept in an environment with a 12 h light/dark cycle with free access to standard chow and water. Mice were randomly divided into four groups: control group, doxorubicin group (Dox group), high-dose *Codonopsis pilosula* polysaccharides group (HCPP group), and low-dose *Codonopsis pilosula* polysaccharides group (LCPP group). After a week of acclimatization, mice in the LCPP and HCPP groups received 100 mg/kg/day and 300 mg/kg/day *Codonopsis pilosula* polysaccharides (KangRuina, LA2667, Beijing, China) by gavage, respectively. The control group and the Dox group were intragastrically administered the same volume of saline. After one week, all three groups received doxorubicin (Solarbio, D8740, Beijing, China) every other day until the cumulative dose reached 24 mg/kg, while the control group received the same amount of saline [49].

### 4.2. Echocardiographic Evaluation

Thoracic echocardiography was performed on anesthetized mice using a Vevo 2100 High-Resolution In Vivo Micro-Imaging System (FUJIFILM VisualSonics, Toronto, ON, Canada). Heart rate was maintained at 450–500 bpm during image acquisition. Left ventricular (LV) echocardiography was performed using parasternal long-axis and short-axis views at a frame rate of 233 Hz. LVIDs, LVESV, FS, EF, SV and CO were calculated based on the left ventricular dimensions at end-systole and end-diastole.

### 4.3. Analysis of Intestinal Microbiota

Fecal samples were collected from mice in each group and stored at −80 °C until analysis. Total genomic DNA was extracted using the CTAB/SDS method. After quality control, 16S rRNA gene sequencing was performed on an Illumina platform by Majorbio Bio-Pharm Technology Co., Ltd. (Shanghai, China), and subsequent bioinformatic analyses were conducted using the Majorbio Cloud Platform. Alpha diversity indices, including Chao1, Shannon, and Simpson, were calculated using mothur software (version 1.30.2), and differences among groups were analyzed using the Kruskal–Wallis test, followed by Dunn’s multiple-comparisons test. Beta diversity was assessed by principal coordinates analysis (PCoA) based on Bray–Curtis distances, and differences in microbial community structure among groups were tested by permutational multivariate analysis of variance (PERMANOVA). Differential bacterial taxa at the genus level were identified by LEfSe analysis, with thresholds of LDA > 2 and *p* < 0.05. Distance-based redundancy analysis (db-RDA) was performed to assess the associations between clinical indicators and gut-microbial community structure. Spearman correlation analysis was used to evaluate the relationships between differential bacterial genera and cardiac/oxidative-stress indices, as well as between differential bacterial genera and predicted KEGG level 3 pathways. The potential functional profiles of the gut microbiota were predicted using PICRUSt (version 2.2.0) based on 16S rRNA sequencing data mapped to the Kyoto Encyclopedia of Genes and Genomes (KEGG) database, and the relative abundances of pathways at KEGG levels 1, 2, and 3 were visualized.

### 4.4. Serological Measurements

After anesthetizing mice with isoflurane (RWD Life Science, R510-22-10, Shenzhen, China), whole blood samples were collected from the eyeballs. The collected blood was transferred into coagulation tubes containing separation gel. Samples were allowed to stand for 3 h and then centrifuged to obtain serum. Serum levels of aspartate aminotransferase (AST), creatine kinase (CK), and lactate dehydrogenase (LDH) were measured using commercial kits (S03040, S03024, and S03034; Rayto, Shenzhen, China). Serum lactate dehydrogenase isoenzyme 1 (LDH-1) and creatine kinase isoenzyme MB (CK-MB) were determined with kits (C058-e and C060; Changchun Huili, Changchun, China). All assays were performed according to the manufacturers’ instructions using the double-reagent method with reagents R1 and R2. Corresponding parameters for each indicator were set on an automated biochemical analyzer, and measurements were obtained by the rate method at a main wavelength of 340 nm and a secondary wavelength of 405 nm. Serum levels of glutathione peroxidase (GSH-PX), malondialdehyde (MDA), and superoxide dismutase (SOD) were measured using assay kits (A005-1, A003-1, and A001-1; Nanjing Jiancheng Bioengineering Institute, Nanjing, China). The assays were conducted according to the manufacturers’ protocols. Briefly, reaction reagents were added as specified, and incubations were carried out under the prescribed temperature and time conditions. Absorbance values were measured using a 1 cm optical path cuvette at 412 nm, 532 nm, and 550 nm. All biochemical parameters were determined strictly in accordance with the instructions provided by the manufacturers.

### 4.5. Histopathology

Hearts were fixed overnight in 4% paraformaldehyde. Heart tissues were dehydrated through a graded ethanol series and subsequent immersion in xylene. Then, tissue was immediately followed by gradual wax immersion through three levels of wax solution. The heart was then embedded in a paraffin embedding machine and frozen for solidification. The resulting wax block was sliced into sections (5 μm). The sections were stained with hematoxylin and eosin (H&E) for routine histological examination under an optical microscope. To assess collagen deposition, selected sections were stained with Sirius Red (G1472, Solarbio, China). For each mouse, three consecutive sections were analyzed, and the fibrotic area was quantified using ImageJ software (version 1.52, National Institutes of Health, Bethesda, MD, USA). Cardiomyocyte cross-sectional area was evaluated by wheat germ agglutinin (WGA) staining (W11261, Thermo Fisher Scientific, Waltham, MA, USA). Histological images were acquired using a Zeiss Axioplan 2 light microscope (Zeiss, Oberkochen, Germany).

### 4.6. Measurements of ATP

The ATP levels in cardiac tissue were determined using an Enhanced ATP Assay Kit (S0027, Beyotime, Beijing, China) according to the manufacturer’s instructions. Briefly, ATP working solution was added to the assay wells on ice, and after the background ATP was completely consumed, the supernatant of the tissue lysate was added. The mixtures were gently mixed, and the relative luminescence units (RLU) were measured using a microplate reader (Thermo Fisher Scientific, Waltham, MA, USA) [50].

### 4.7. Real-Time Quantitative PCR (qPCR)

According to the manufacturer’s protocol, total RNA was extracted from mouse heart tissue using TRIzol reagent, followed by reverse transcription with a commercial reverse transcription kit (11141S60, Yeasen, Shanghai, China). The resulting cDNA was aliquoted and used as the template for quantitative PCR (qPCR) analysis. β-Actin served as the internal reference gene. The primer sequences used for qPCR are listed in Table S1. Gene expression levels were quantified using the 2^–ΔΔCT method.

### 4.8. Transmission Electron Microscopy

Hearts were cut into 1–2 mm^3^ tissue blocks and fixed for 2 h at room temperature in 2.5% glutaraldehyde prepared in 0.1 M sodium cacodylate buffer (pH 7.4). The samples were then stored overnight at 4 °C, post-fixed and embedded in resin, and ultrathin sections (~90 nm) were obtained using an ultramicrotome. Sections were examined with a Hitachi HT7800 transmission electron microscope (Hitachi, Tokyo, Japan), and images were captured using a 2k slow-scan CCD camera (Gatan, Pleasanton, CA, USA).

### 4.9. Physicochemical Characterization of the Codonopsis pilosula Polysaccharide Preparation

The structural features of CPP were analyzed by Fourier-transform infrared (FT-IR) spectroscopy, high-performance liquid chromatography (HPLC), and gel permeation chromatography (GPC).

For FT-IR analysis, CPP powder was mixed with dry KBr, pressed into pellets, and scanned using an FT-IR spectrophotometer (Thermo Fisher Scientific, Waltham, MA, USA, 4000–400 cm^−1^ range). Characteristic absorption peaks were recorded to identify functional groups, such as hydroxyl (–OH), C–H, and glycosidic (C–O–C) bonds. The monosaccharide composition of CPP was determined by HPLC after acid hydrolysis and derivatization with 1-phenyl-3-methyl-5-pyrazolone (PMP). The mixture was separated on a C18 column with a mobile phase of phosphate buffer and acetonitrile, and monosaccharides were identified by comparing retention times with standard sugars.

The apparent molecular-weight distribution of CPP was analyzed by gel permeation chromatography/size-exclusion chromatography (GPC/SEC) using a refractive-index detector. Molecular-weight calibration was performed with pullulan standards, and the number-average molecular weight (Mn), weight-average molecular weight (Mw), Z-average molecular weight (Mz), and dispersity (Mw/Mn) were calculated using Shimadzu LabSolutions GPC/SEC software (version 5.54, Shimadzu Corporation, Kyoto, Japan). The reported molecular-weight values are therefore pullulan-equivalent apparent values.

### 4.10. Statistical Analysis

Statistical analyses were performed using GraphPad Prism 9.5 (GraphPad Software, San Diego, CA, USA). Data are presented as mean ± standard deviation (SD), unless otherwise indicated. For comparisons among multiple groups, one-way analysis of variance (ANOVA), followed by Tukey’s multiple-comparisons test, was used. For gut-microbiota analysis, alpha diversity indices and the relative abundances of selected genera were compared using the Kruskal–Wallis test, followed by Dunn’s multiple-comparisons test. Beta diversity was assessed by principal coordinates analysis (PCoA) based on Bray–Curtis distances, and group differences were tested by PERMANOVA. Correlations were analyzed using Spearman’s rank correlation test. Survival was analyzed by the Kaplan–Meier method with the log-rank test. A two-sided *p* < 0.05 was considered statistically significant.

## Data Availability

The raw data supporting the conclusions of this article will be made available by the authors on request.

## Supplementary Materials

The following supporting information can be downloaded at: https://www.mdpi.com/article/10.3390/ijms27156973/s1.

## References

[1] Kong C.Y., Guo Z., Song P., Zhang X., Yuan Y.P., Teng T., Yan L., Tang Q.Z. (2022). Underlying the mechanisms of doxorubicin-Induced acute cardiotoxicity: Oxidative stress and cell death. Int. J. Biol. Sci..

[2] L’Ecuyer T., Sanjeev S., Thomas R., Novak R., Das L., Campbell W., Heide R.V. (2006). DNA damage is an early event in doxorubicin-induced cardiac myocyte death. Am. J. Physiol. Heart Circ. Physiol..

[3] Cappetta D., De Angelis A., Sapio L., Prezioso L., Illiano M., Quaini F., Rossi F., Berrino L., Naviglio S., Urbanek K. (2017). Oxidative stress and cellular response to doxorubicin: A common factor in the complex milieu of anthracycline cardiotoxicity. Oxidative Med. Cell. Longev..

[4] Tokarska-Schlattner M., Zaugg M., Zuppinger C., Wallimann T., Schlattner U. (2006). New insights into doxorubicin-induced cardiotoxicity: The critical role of cellular energetics. J. Mol. Cell. Cardiol..

[5] Hasinoff B.B., Herman E.H. (2007). Dexrazoxane: How it works in cardiac and tumor cells. Is it a prodrug or is it a drug?. Cardiovasc. Toxicol..

[6] Swain S.M., Whaley F.S., Ewer M.S. (2003). Congestive heart failure in patients treated with doxorubicin: A retrospective analysis of three trials. Cancer.

[7] Guo J., Guo Q., Fang H., Lei L., Zhang T., Zhao J., Peng S. (2014). Cardioprotection against doxorubicin by metallothionein is associated with preservation of mitochondrial biogenesis involving PGC-1α pathway. Eur. J. Pharmacol..

[8] Kavazis A.N., Morton A.B., Hall S.E., Smuder A.J. (2017). Effects of doxorubicin on cardiac muscle subsarcolemmal and intermyofibrillar mitochondria. Mitochondrion.

[9] Dorn G.W., Vega R.B., Kelly D.P. (2015). Mitochondrial biogenesis and dynamics in the developing and diseased heart. Genes Dev..

[10] Zhang S., Liu X., Bawa-Khalfe T., Lu L.S., Lyu Y.L., Liu L.F., Yeh E.T. (2012). Identification of the molecular basis of doxorubicin-induced cardiotoxicity. Nat. Med..

[11] Lipshultz S.E., Scully R.E., Lipsitz S.R., Sallan S.E., Silverman L.B., Miller T.L., Barry E.V., Asselin B.L., Athale U., Clavell L.A. (2010). Assessment of dexrazoxane as a cardioprotectant in doxorubicin-treated children with high-risk acute lymphoblastic leukaemia: Long-term follow-up of a prospective, randomised, multicentre trial. Lancet Oncol..

[12] Reichardt P., Tabone M.D., Mora J., Morland B., Jones R.L. (2018). Risk-benefit of dexrazoxane for preventing anthracycline-related cardiotoxicity: Re-evaluating the European labeling. Future Oncol..

[13] Tebbi C.K., London W.B., Friedman D., Villaluna D., De Alarcon P.A., Constine L.S., Mendenhall N.P., Sposto R., Chauvenet A., Schwartz C.L. (2007). Dexrazoxane-associated risk for acute myeloid leukemia/myelodysplastic syndrome and other secondary malignancies in pediatric Hodgkin’s disease. J. Clin. Oncol..

[14] Cao L.Y., Li X.X., Wang D., Sun H.F., Gao J.P. (2017). Validation of reliable reference genes for accurate normalization in RT-qPCR analysis of *Codonopsis pilosula*. Chin. Herb. Med..

[15] Wang F., Yang Y., Wang L., Wang H., Wang W., Yang S., Yang M., Zhang C., Wang Y. (2025). Purified *C. pilosula* polysaccharides attenuate streptozotocin-induced oxidative stress and restore gut microbiota in T2DM mice. Int. J. Biol. Macromol..

[16] Chen D., Liu J., Meng J., Li D., Zhao P., Duan Y., Wang J. (2020). Integrative analysis of long non-coding RNAs (lncRNAs), miRNAs, and mRNA-associated ceRNA network in lung tissue of aging mice and changes after treatment with *Codonopsis pilosula*. Med. Sci. Monit..

[17] Guo X.N., Qi H.Y., Wang B., Dong J.L., Jiang J., Yang Y.J. (2013). Anti-aging effects of polysaccharide from *Codonopsis pilosula* on the model of aging mice. Chin. J. Gerontol..

[18] Agus A., Clément K., Sokol H. (2021). Gut microbiota-derived metabolites as central regulators in metabolic disorders. Gut.

[19] Liu J., Tan Y., Cheng H., Zhang D., Feng W., Peng C. (2022). Functions of Gut Microbiota Metabolites, Current Status and Future Perspectives. Aging Dis..

[20] Cao L., Du C., Zhai X., Li J., Meng J., Shao Y., Gao J. (2022). *Codonopsis pilosula* polysaccharide improved spleen deficiency in mice by modulating gut microbiota and energy related metabolisms. Front. Pharmacol..

[21] Fu J., Chang C., Ren J., Cheng J., Gao Z., Meng Y. (2025). The Structure characterization of polysaccharides from codonopsis pilosula and the structure-activity relationship with immune-regulation on THP-1 Cells. Chem. Biodivers..

[22] Hong T., Yin J.Y., Nie S.P., Xie M.Y. (2021). Applications of infrared spectroscopy in polysaccharide structural analysis: Progress, challenge and perspective. Food Chem. X.

[23] Li M., Zhang R., Li X., Huang H. (2026). The Metabolic Role of Mitochondria in the Perinatal Cardiac Development and Cardiovascular Diseases. Exploration.

[24] Guan T., Song J., Wang Y., Guo L., Yuan L., Zhao Y., Gao Y., Lin L., Wang Y., Wei J. (2017). Expression and characterization of recombinant bifunctional enzymes with glutathione peroxidase and superoxide dismutase activities. Free Radic. Biol. Med..

[25] Ma Y.L., Kong C.Y., Guo Z., Wang M.Y., Wang P., Liu F.Y., Yang D., Yang Z., Tang Q.Z. (2024). Semaglutide ameliorates cardiac remodeling in male mice by optimizing energy substrate utilization through the Creb5/NR4a1 axis. Nat. Commun..

[26] Yang Y., Owusu F.B., Wu H., Zhang X., Li R., Liu Z., Zhang S., Leng L., Wang Q. (2025). Mitochondria as therapeutic targets for natural products in the treatment of cardiovascular diseases. J. Ethnopharmacol..

[27] Zhou Y., Ni Z., Liu J., Sun D., Shen P., Chen X., Li G., Bai Z., Hu Y., Wang N. (2025). Gut Microbiota-Associated Metabolites Affected the Susceptibility to Heart Health Abnormality in Young Migrants at High-Altitude. Exploration.

[28] Sun S., Yang S., Dai M., Jia X., Wang Q., Zhang Z., Mao Y. (2017). The effect of Astragalus polysaccharides on attenuation of diabetic cardiomyopathy through inhibiting the extrinsic and intrinsic apoptotic pathways in high glucose -stimulated H9C2 cells. BMC Complement. Altern. Med..

[29] Xu X., Rui S., Chen C., Zhang G., Li Z., Wang J., Luo Y., Zhu H., Ma X. (2020). Protective effects of astragalus polysaccharide nanoparticles on septic cardiac dysfunction through inhibition of TLR4/NF-κB signaling pathway. Int. J. Biol. Macromol..

[30] Li Y., Zhang W. (2022). Effect of ginsenoside rb2 on a myocardial cell model of coronary heart disease through Nrf2/HO-1 signaling pathway. Biol. Pharm. Bull..

[31] Cui Y., Wu J., Wang Y., Li D., Zhang F., Jin X., Li M., Zhang J., Liu Z. (2024). Protective effects of ginsenoside F2 on isoproterenol-induced myocardial infarction by activating the Nrf2/HO-1 and PI3K/Akt signaling pathways. Phytomedicine.

[32] Li H., Zhang L., Li X., He H., Fu G., Zhu Y.Z., Hu W., Qiu L., Gong L., Zhang Y. (2025). Improvement on mitochondrial energy metabolism of *Codonopsis pilosula* (Franch.) *Nannf.* polysaccharide. Front. Pharmacol..

[33] Song J., Luo X., Xiong L., Li S., Chen Y., Liu W., Yuan Y., Ma Y., Bian J., Liu Z. (2025). *Lycium barbarum* polysaccharide alleviate cadmium-induced mitochondrial dysfunction mediated pyroptosis in duck liver. Int. J. Biol. Macromol..

[34] Chen S., Li Q., Shi H., Li F., Duan Y., Guo Q. (2024). New insights into the role of mitochondrial dynamics in oxidative stress-induced diseases. Biomed. Pharmacother..

[35] Kowalczyk P., Sulejczak D., Kleczkowska P., Bukowska-Ośko I., Kucia M., Popiel M., Wietrak E., Kramkowski K., Wrzosek K., Kaczyńska K. (2021). Mitochondrial oxidative stress—A causative factor and therapeutic target in many diseases. Int. J. Mol. Sci..

[36] Pietrangelo D., Lopa C., Litterio M., Cotugno M., Rubattu S., Lombardi A. (2025). Metabolic disturbances involved in cardiovascular diseases: The role of mitochondrial dysfunction, altered bioenergetics and oxidative stress. Int. J. Mol. Sci..

[37] Singh P., Sharma R., McElhanon K., Allen C.D., Megyesi J.K., Beneš H., Singh S.P. (2015). Sulforaphane protects the heart from doxorubicin-induced toxicity. Free Radic. Biol. Med..

[38] Liu D., Ma Z., Di S., Yang Y., Yang J., Xu L., Reiter R.J., Qiao S., Yuan J. (2018). AMPK/PGC1α activation by melatonin attenuates acute doxorubicin cardiotoxicity via alleviating mitochondrial oxidative damage and apoptosis. Free Radic. Biol. Med..

[39] Song J.H., Kim M.S., Lee S.H., Hwang J.T., Park S.H., Park S.W., Jeon S.B., Lee R.R., Lee J., Choi H.K. (2024). Hydroethanolic extract of *Cirsium setidens* ameliorates doxorubicin-induced cardiotoxicity by AMPK-PGC-1α-SOD-mediated mitochondrial protection. Phytomedicine.

[40] Luna-López A., González-Puertos V.Y., López-Diazguerrero N.E., Königsberg M. (2014). New considerations on hormetic response against oxidative stress. J. Cell Commun. Signal..

[41] Yuan H., Ma Q., Cui H., Liu G., Zhao X., Li W., Piao G. (2017). How can synergism of traditional medicines benefit from network pharmacology?. Molecules.

[42] Tu Y., Dai G., Chen Y., Tan L., Liu H., Chen M. (2025). Emerging target discovery strategies drive the decoding of therapeutic power of natural products and further drug development: A case study of celastrol. Exploration.

[43] An L., Wuri J., Zheng Z., Li W., Yan T. (2021). Microbiota modulate Doxorubicin induced cardiotoxicity. Eur. J. Pharm. Sci..

[44] Huang C., Li X., Li H., Chen R., Li Z., Li D., Xu X., Zhang G., Qin L., Li B. (2024). Role of gut microbiota in doxorubicin-induced cardiotoxicity: From pathogenesis to related interventions. J. Transl. Med..

[45] Fu Y.P., Feng B., Zhu Z.K., Feng X., Chen S.F., Li L.X., Yin Z.Q., Huang C., Chen X.F., Zhang B.Z. (2018). The Polysaccharides from *Codonopsis pilosula* Modulates the Immunity and Intestinal Microbiota of Cyclophosphamide-Treated Immunosuppressed Mice. Molecules.

[46] Sansonetti P.J. (2001). Rupture, invasion and inflammatory destruction of the intestinal barrier by Shigella, making sense of prokaryote-eukaryote cross-talks. FEMS Microbiol. Rev..

[47] Zhu Y., Chen B., Zhang X., Akbar M.T., Wu T., Zhang Y., Zhi L., Shen Q. (2024). Exploration of the Muribaculaceae Family in the Gut Microbiota: Diversity, Metabolism, and Function. Nutrients.

[48] Yang Y., Song X., Wang G., Xia Y., Xiong Z., Ai L. (2024). Understanding *Ligilactobacillus salivarius* from Probiotic Properties to Omics Technology: A Review. Foods.

[49] Wan S., Qi J., Xia Y., Fan C., Xu T., Zhang X., Shi J., Wang C., Cheng Y., Zhang D. (2025). Cardiac Slc25a49-mediated energy reprogramming governs doxorubicin-Induced cardiomyopathy through the G6P-AP-1-Sln Axis. Adv. Sci..

[50] Li Y., Ye Z., Lai W., Rao J., Huang W., Zhang X., Yao Z., Lou T. (2017). Activation of sirtuin 3 by silybin attenuates mitochondrial dysfunction in cisplatin-induced acute kidney injury. Front. Pharmacol..

