# Supplementary material for: A Heterogeneous Codonopsis pilosula Polysaccharide Preparation Attenuates Doxorubicin-Induced Cardiotoxicity in Mice: Associations with Oxidative Stress, Mitochondrial Function, and Gut Microbiota
Source: Int J Mol Sci. 2026 Aug 3;27(15):6973. doi: 10.3390/ijms27156973 (PMC13466922; doi:10.3390/ijms27156973)
Supplement: Supplementary file 1 [file ijms-27-06973-s001.zip › ijms-4468949-supplementary.pdf]

Table S1. Primers used for the PCR assays in this study.

Gene	GeneBank sequence number	Forward primer (5'->3')	Reverse Primer (5'->3')
Anp	NM_008725	GCTTCGGGGGTAGGATTGAC	GAGGCAAGACCCCACTAGAC
Bnp	NM_008726	GGAAGTCCTAGCCAGTCTCCAGAG	GCCTTGGTCCTTCAAGAGCTGTC
Col1a1	NM_007742	CCTCAGGGTATTGCTGGACAAC	CAGAAGGACCTTGTTTGCCAGG
Col5a1	NM_015734	CTTCGCCGCTACTCCTGTTC	CCCTGAGGGCAAATTGTGAAAA
Ndufb8	NM_026061	CGCCAAGAAGTATAACATGCGAG	CCTCTCATGCTGTGATCGGTTG
Sdhb	NM_023374	TGCGGACCTATGGTGTTGGATG	CCAGAGTATTGCCTCCGTTGATG
Uqcc1	NM_018888	CAGCCTGTTGAAGAGAAGGTCG	CGAAGTCAGTCTTCTCCACGCA
Cox5b	NM_009941	TGGTGCTGGTGGTAGAGTGT	AGGGTGATGTTGAGGTTGGC
Atp5f1a	NM_007505	TGGTGAAGAGACTGACGGATGC	TCAAAGCGTGCTTGCCGTTGTC
Mmp2	NM_008610	CAAGGATGGACTCCTGGCACAT	CCGACCGTTGAACAGGAAGG
Actb	NM_007393	GGTACCACCATGTACCCAGG	AAACGCAGCTCAGTAACAGTC
